# Supplementary figures and images for: Elastic Free Energy Drives the Shape of Prevascular Solid Tumors
Source: PLoS One. 2014 Jul 29;9(7):e103245. doi: 10.1371/journal.pone.0103245 (PMC4114546; doi:10.1371/journal.pone.0103245)

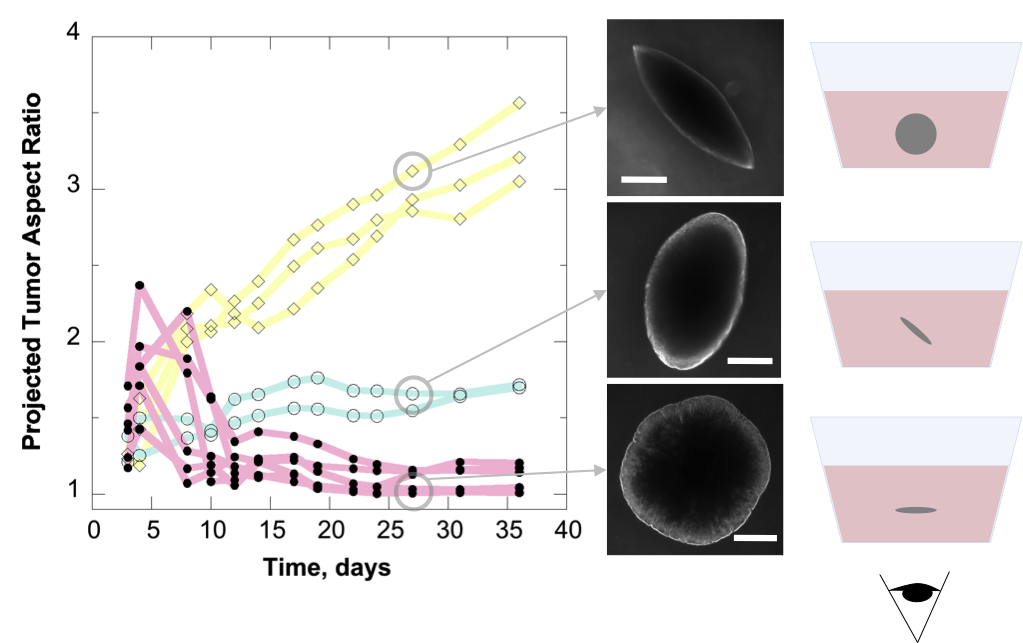

Supplement: Figure S1 — Individual tumor development. To determine the time frame over which the oblate ellipsoidal shape developed, individually embedded, pre-produced tumor spheroids were followed as they grew in the agarose gel. In such an experiment, it was determined that within one week after embedment the ellipsoidal shape was detectable and the orientation of the tumor in the gel was fixed. Shown here is the time course development of the projected tumor aspect ratio of 11 different individual tumors (sparsely embedded). Diamond symbols mark the aspect-ratio measurements for tumors whose final orientation projected narrow ellipses, solid circles for tumors whose final orientation projected near-circles, and open circles for tumors whose final orientation projected wider ellipses. Phase-contrast images show the projections from which the aspect ratios were measured for the specific tumors and time points circled in grey on the plot. Scale bars are 500 µm. (TIFF) [file pone.0103245.s001.tiff]

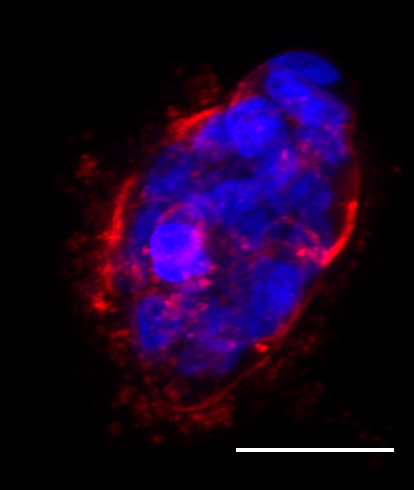

Supplement: Figure S2 — In the ellipsoidal cross-section of a three-day-old tumor, a necrotic core is not detectable. It was confirmed that the tumors took on the oblate ellipsoidal shape before the observation of a central necrotic core, ruling out the possibility that this shape formed due to mechanical collapse of a necrosed core. Three-dimensional projection of 13 image slices, taken 2 µm apart in the mid-section of an oblate ellipsoidal tumor. The section is shown here tilted about the vertical axis of the image by 10 degrees. Nuclei (blue) and actin network (red)of LS174T cells embedded in 1% agarose hydrogel. Scale bar is 30 µm. (TIF) [file pone.0103245.s002.tif]

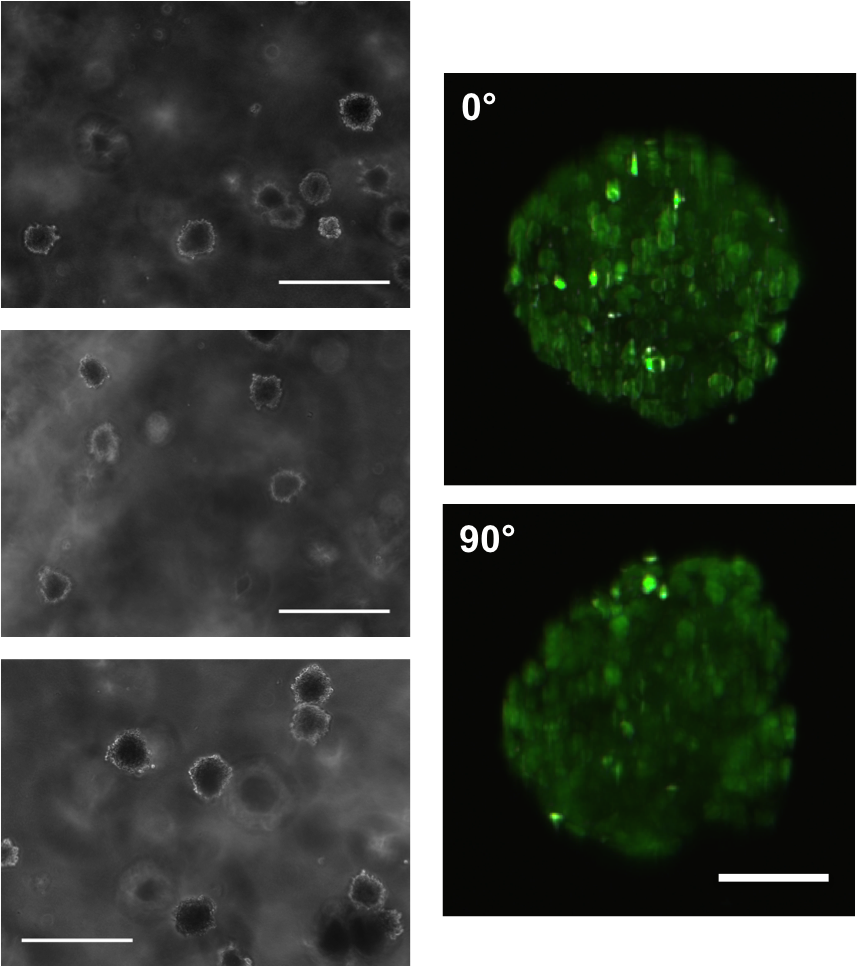

Supplement: Figure S3 — Tumors grown in 0.3% agarose. Left Column: Phase contrast images of 18-day-old tumors taken with a 5x objective. Scale bar is 500 µm. Right Column: Projections at two perpendicular orientations of a 15-day-old tumor. The fluorescent signal is from the Hoechst-stained nuclei. The scale bar is 50 µm. The tumor shape in the softest gels is oftenmore diffuse and approximately spherical. (TIFF) [file pone.0103245.s003.tiff]

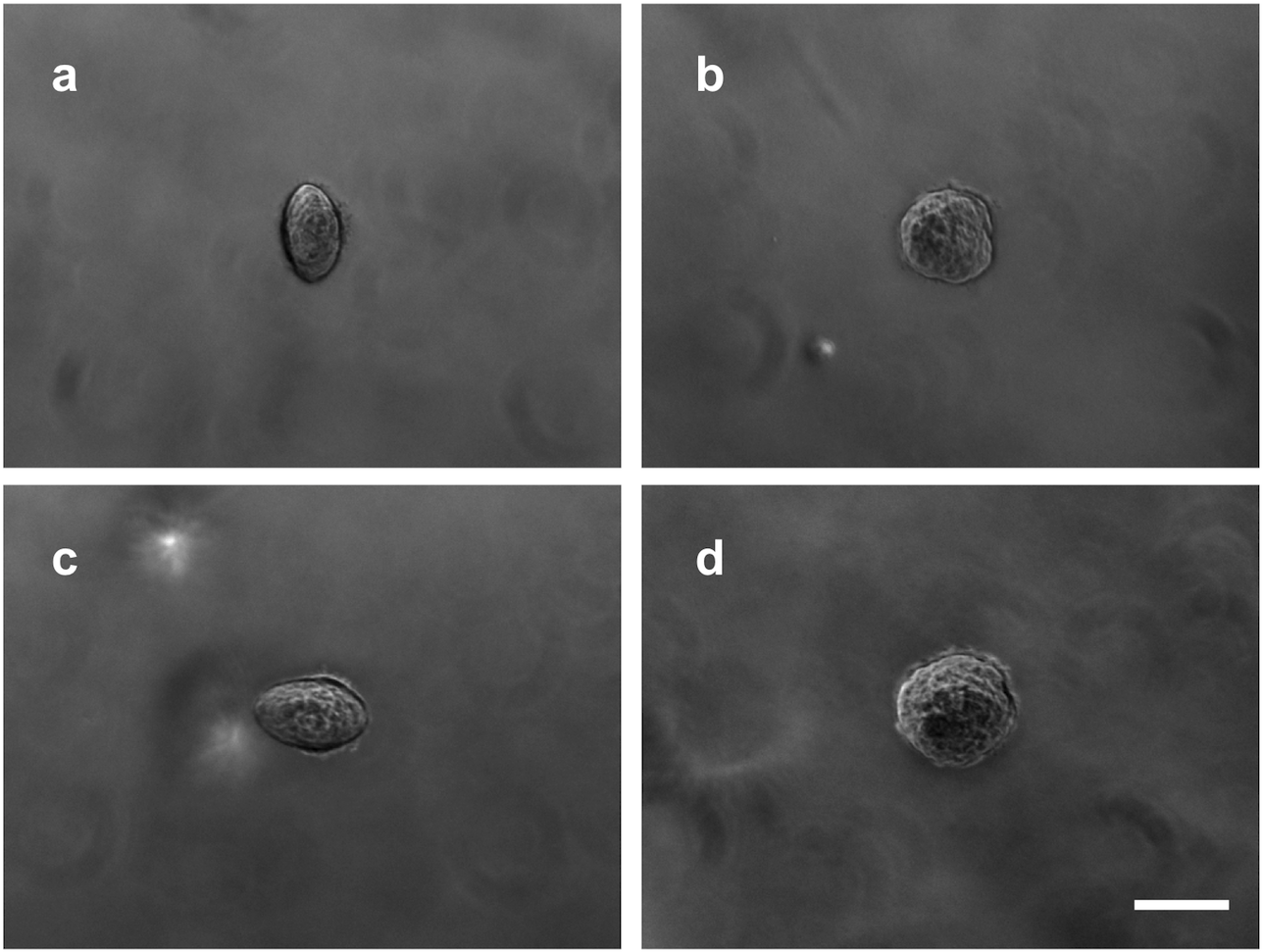

Supplement: Figure S4 — Phase-contrast images of the projections of four different tumors grown for 9 days from HeLa cervix adenocarcinoma cells embedded in 0.5% agarose hydrogels. Elliptical and circular cross-sections, similar to those seen from tumors grown from LS174T cells, are observed. The scale bar is 100 µm. (TIF) [file pone.0103245.s004.tif]

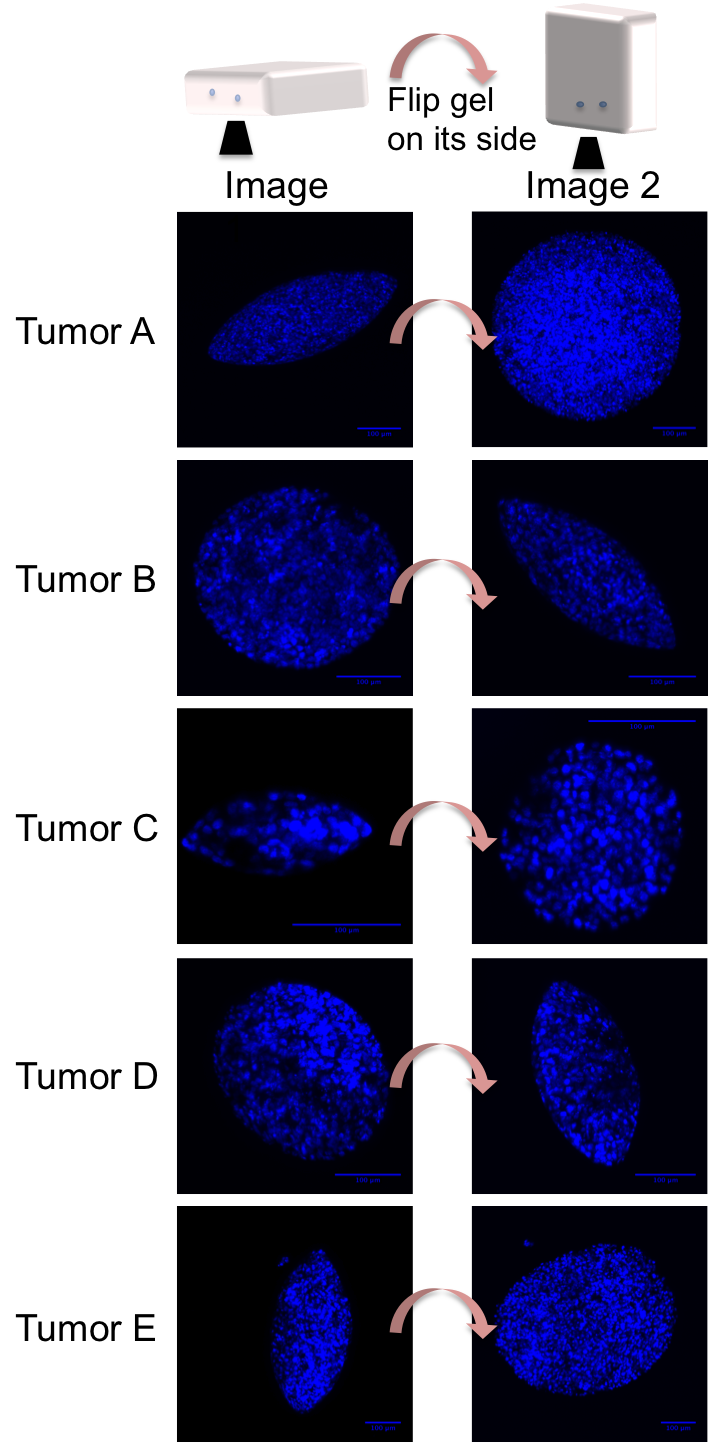

Supplement: Figure S5 — Perpendicular views of 5 tumors grown in 1% agarose. Due to scattering regular confocal microscopy is not effective at capturing entire 3D tumor shapes. However, by carefully sectioning the gel in the region of a tumor, two approximately perpendicular views may be imaged of the tumors. Here are some examples. The fluorescent signal is provided by Hoechst-stained nuclei. The scale bars are 100 µm long. (TIFF) [file pone.0103245.s005.tiff]
