# Supplementary material for: Elastic Free Energy Drives the Shape of Prevascular Solid Tumors
Source: PLoS One. 2014 Jul 29;9(7):e103245. doi: 10.1371/journal.pone.0103245 (PMC4114546; doi:10.1371/journal.pone.0103245)
Supplement: File S1 — The Supporting Information file contains the supporting text for the statistical testing on measured 3D tumor dimensions, the outline of the proof for the projection of an oblate ellipsoid always containing its major axes, and further details on our observation that the hydrogel did not crack due to tensile normal stress components induced tangential to the tumor-gel boundary. The Supporting Information file also contains Figures S6 and S7 and Tables S1 and S2. (DOCX) [file pone.0103245.s006.docx]

Elastic free energy drives the shape of prevascular solid tumors

Supporting Information

K. L. Mills, Ralf Kemkemer, Shiva Rudraraju, and Krishna Garikipati

correspondence to: mills@is.mpg.de or krishna@umich.edu

**Table of Contents**

**Statistical testing on measured 3D tumor dimensions** 2

Supporting Table 1: Three-dimensional tumor measurements

Supporting Table 2: Statistical test values

**The projection of an oblate ellipsoid always contains its major axes** 3

Supporting Figure 6: Three examples of a plane intersecting an oblate ellipsoid

**Crack initiation in the agarose hydrogels** 4

Supporting Figure 7: Cracks in the gel are not associated with tumor growth

**Author Contributions** 5

Statistical testing on measured 3D tumor dimensions

|  |  |  | **Tumor diameter, μm** | | |
| --- | --- | --- | --- | --- | --- |
|  |  |  | **2a_1_** | **2a_2_** | **2a_3_** |
| **Tumor designation** | **0.5%** | **1) Fig. 2 Tumor 1** | 289 | 248 | 117 |
|  |  | **2) Fig. 2 Tumor 2** | 247 | 227 | 114 |
|  |  | **3) R005** | 91 | 74 | 39 |
|  | **1.0%** | **4) Fig. 1** | 371 | 358 | 138 |
|  |  | **5) R002** | 207 | 183 | 80 |
|  |  | **6) R004** | 514 | 507 | 157 |
|  |  | **7) Fig. S5 Tumor A** | 445 | 442 | 140 |
|  |  | **8) Fig. S5 Tumor B** | 315 | 308 | 123 |
|  |  | **9) Fig. S5 Tumor C** | 179 | 176 | 74 |
|  |  | **10) Fig. S5 Tumor D** | 283 | 264 | 120 |
|  |  | **11) Fig. S5 Tumor E** | 517 | 427 | 181 |

**Table S1.** 3D measurements of the tumor axes 2*a*_1_, 2*a*_2_ and 2*a*_3_ from 11 different tumors in 0.5% and 1.0% agarose hydrogels. The second column is the tumor designation, which, when included in the paper or this Supplementary Materials document, is referred to by its caption number.

Definitions of Null Hypotheses:

*The tumor shape is not oblate ellipsoidal*

(a) **1)** difference between *a*_1_ and *a*_2_, (*a*_1_-*a*_2_)/*a*_1_, is greater than 0.15 (μ_0,1_ > 0.15) AND

**2)** oblateness, , is less than 0.5 (μ_0,2_ < 0.5) (i.e., the long axes are less than 2 times as long as the short axis)

(b) **3)** *a*_1_/*a*_3_ < 2 (μ_0,3_ < 2) AND

**4)** *a*_2_ is closer to *a*_3_ than to *a*_1_, (*a*_1_-*a*_2_)/(*a*_2_-*a*_3_) >1 (μ_0,4_ > 1)

*The tumor shape is a spheroid*

**5)** no difference between *a*_1_ and *a*_2_ (μ_0,5_ = 0) AND

**6)** oblateness, *f*, is equal to 0 (μ_0,6_ = 0)

*The tumor shape is a general ellipsoid (a_2_ is half-way between a_1_ and a_3_)*

**7)** difference between *a*_1_ and *a*_2_, (*a*_1_-*a*_2_)/*a*_1_ is 0.5 (μ_0_,*_7_* = 0.5) AND

**8)** oblateness, *f*, is 0.75 (μ_0,8_ = 0.75)

|  |  | Average, $\bar{x}$ | Standard deviation, σ |  | p-value  (*two-tailed) |
| --- | --- | --- | --- | --- | --- |
| **Null Hypothesis, n_i_** | **1)** μ_0,1_ > 0.15 | 0.079 | 0.068 | -3.493 | 0.003 |
|  | **2)** μ_0,2_ < 0. 5 | 0.597 | 0.057 | 5.635 | 0.00018 |
|  | **3)** μ_0,3_ < 2 | 2.629 | 0.354 | 5.888 | 0.000077 |
|  | **4)** μ_0,4_ > 1 | 0.171 | 0.163 | -16.898 | 5.5 × 10^-9^ |
|  | **5)** μ_0,5_ = 0 | 0.079 | 0.068 | 3.841 | 0.003* |
|  | **6)** μ_0,6_ = 0 | 0.597 | 0.057 | 34.724 | 9.3 × 10^-12^* |
|  | **7)** μ_0,7_ = 0.5 | 0.079 | 0.068 | -20.605 | 1.6 × 10^-9^* |
|  | **8)** μ_0,8_ = 0.75 | 0.597 | 0.057 | -8.909 | 4.5 × 10^-6^* |

**Table S2.** Statistical test values, by a Student’s T-test, for the null hypotheses listed above. The Bonferroni adjustment gives individual significance values for each test of 0.00625 when a total significance value of 0.05 is considered.

The projection of an oblate ellipsoid always contains its major axes

The projection of an oblate ellipsoid is the ellipse that results from the intersection of a plane—oriented normal to the projection direction—as it is passed through the oblate ellipsoid. When this plane shares the centroid with the oblate ellipsoid (Fig. S6), the major axis of the ellipse will be equal in length to the major axes (2*a*_1_ = 2*a*_2_) of the oblate ellipsoid, no matter what the projection direction is. Since no other intersection contains a larger axis, this means that the major axis of any projection of an oblate ellipsoid is equal to the major axes of the oblate ellipsoid.


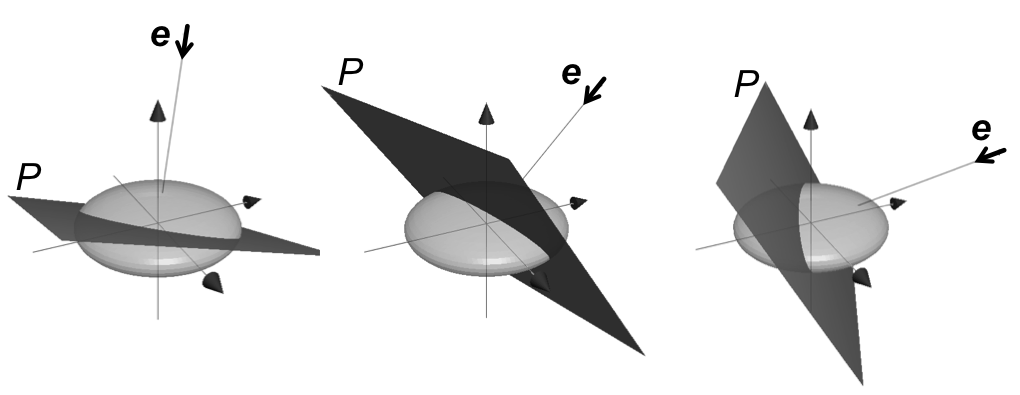


**Figure S6.** Three examples of a plane intersecting an oblate ellipsoid. The direction of projection, with unit vector, $\boldsymbol{e}$, is drawn from an arbitrary point of observation through the centroid of the oblate ellipsoid. The plane, $P$, is normal to $\boldsymbol{e}$ and contains the centroid of the oblate ellipsoid. The ellipse that is created by the intersection of any such plane with the oblate ellipsoid has as its larger axis the major axes of the oblate ellipsoid: ${2a}_{1}={2a}_{2}$.

Crack initiation in the agarose hydrogels

The growth strain additionally leads to a normal stress in the gel tangential to the tumor-gel boundary: *σ_33_* along the *x_1_* direction and *σ_11_* along the *x_3_* direction (26). Notably, these are discontinuous at the tumor-gel boundary, going from compressive within the tumor to tensile in the gel.

Cheng and co-workers (7) suggested that the tensile *σ_33_* stress at the tip of the major axis of the oblate ellipsoid induces cracking of the agarose hydrogel. We have not found such cracks in this study (Fig. S7a). More often, cracks in the gel, which take on an ellipsoidal cross-section in the plane of the cell culture well, were seen immediately following agarose gelation; and, at later time points in the experiment, they were not associated with a tumor (Fig. S7b).

The cracks were sometimes associated with bubbles that were incorporated in the agarose before gelation. When not associated with a bubble, the cracks may have formed during the gelation process itself. If liquid evaporates during this time, the gel will contract. Since the gel is bonded to the walls of the cell-culture well, a tensile stress would develop in the agarose hydrogel in the plane of the cell culture well. Depending on its magnitude, the stress may be large enough to produce a crack in the gel in the orientation that is experimentally observed.

**
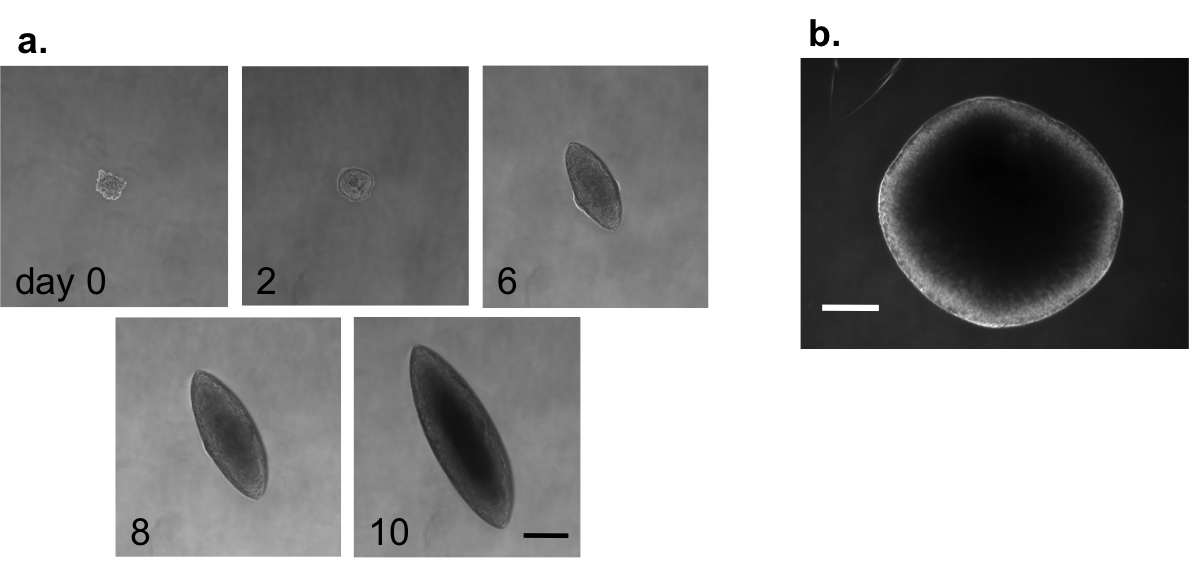
**

**Figure S7. Cracks in the gel are not associated with tumor growth. (a)** An oblate ellipsoidal tumor with its minor axis (*a_3_*) oriented in the projection view grows from a major diameter of 160 mm and aspect ratio of 1.2 to a major diameter of 920 μm and aspect ratio of 2.8 over the course of 10 days. The scale bar is 200 μm. **(b)** Above and to the left of the tumor in this image, the bottom half of a crack in the hydrogel is visible that is not associated with a tumor growing inside of it. Note that the hydrogel is cleaved to the upper left of the crack boundary in this image. The scale bar is 500 μm.

**Author Contributions**

KLM, KG, and RK conceived the research and designed the experiments. KLM performed the experiments. KLM analyzed and KLM, KG, and RK interpreted the data. KLM and KG performed the elasticity calculations. KG and SR performed the computational modeling. KLM and KG wrote the paper.
